# Mathematical modelling of phenotypic plasticity and conversion to a stem-cell state under hypoxia

**DOI:** 10.1038/srep18074

**Published:** 2016-02-03

**Authors:** Andrew Dhawan, Seyed Ali Madani Tonekaboni, Joseph H. Taube, Stephen Hu, Nathalie Sphyris, Sendurai A. Mani, Mohammad Kohandel

**Affiliations:** 1 School of Medicine, Queen’s University, Kingston, Ontario, Canada; 2 Department of Applied Mathematics, University of Waterloo, Waterloo, Ontario, Canada; 3 Department of Translational Molecular Pathology, The University of Texas MD Anderson Cancer Centre, Houston, Texas, United States of America; 4 Metastasis Research Centre, The University of Texas MD Anderson Cancer Centre, Houston, Texas, United States of America; 5 Centre for Mathematical Medicine, Fields Institute for Research in Mathematical Sciences, Toronto, Ontario, Canada

## Abstract

Hypoxia, or oxygen deficiency, is known to be associated with breast tumour progression, resistance to conventional therapies and poor clinical prognosis. The epithelial-mesenchymal transition (EMT) is a process that confers invasive and migratory capabilities as well as stem cell properties to carcinoma cells thus promoting metastatic progression. In this work, we examined the impact of hypoxia on EMT-associated cancer stem cell (CSC) properties, by culturing transformed human mammary epithelial cells under normoxic and hypoxic conditions, and applying *in silico* mathematical modelling to simulate the impact of hypoxia on the acquisition of CSC attributes and the transitions between differentiated and stem-like states. Our results indicate that both the heterogeneity and the plasticity of the transformed cell population are enhanced by exposure to hypoxia, resulting in a shift towards a more stem-like population with increased EMT features. Our findings are further reinforced by gene expression analyses demonstrating the upregulation of EMT-related genes, as well as genes associated with therapy resistance, in hypoxic cells compared to normoxic counterparts. In conclusion, we demonstrate that mathematical modelling can be used to simulate the role of hypoxia as a key contributor to the plasticity and heterogeneity of transformed human mammary epithelial cells.

The tumour microenvironment has long been recognized as a key factor driving the tumour growth and metastasis underpinning breast cancer progression^1^. The tumour microenvironment is a dynamic, complex and continually evolving entity that forms the stroma surrounding the tumour, and is composed of multiple cell types (fibroblasts, myoepithelial cells, endothelial cells, various infiltrating immune cell types) as well as the extracellular matrix, inflammatory cytokines, growth factors and environmental stresses. Indeed, the microenvironment at the invasive edge of the tumour differs dramatically from that of the tumour core.

A key feature of the tumour microenvironment is the presence of hypoxia^2^, which results from an imbalance between the oxygen supply and demand, leading to localised oxygen deprivation in some regions of the tumour. Specifically, within solid tumours, hypoxia arises due to the inadequate supply of oxygen in relation to the high rates of cell proliferation combined with an inefficient and ineffective vascular supply^3^. Most tumours—larger than 1 mm^3^—contain hypoxic regions as a result of the increased oxygen diffusion distances and the disordered and inadequate tumour vasculature, adding biochemical and metabolic stresses to the tumour cells^3^. Overall, hypoxia tends to be associated with the core of the tumour, whereas oxygen is more freely available at the periphery/invasive edge.

Hypoxia impacts tumour growth in multiple respects e.g. by stimulating recruitment of inflammatory cell types and endothelial cells, enhancing angiogenesis, promoting immune suppression, exacerbating inflammation and supporting metabolic reprogramming^4^. In addition, hypoxic conditions serve to confer a selective pressure for the preferential survival of resilient stem-like tumour cells that promote metastatic dissemination^2^,^4^. In this regard, hypoxia has been implicated in the induction of a process, known as the epithelial-mesenchymal transition (EMT), that has been shown to confer intrinsic migratory and invasive capabilities as well as stem cell properties to carcinoma cells, thus enhancing their metastatic potential^2^,^5^,^6^. Upon undergoing EMT, epithelial cells typically acquire a less differentiated mesenchymal morphology characterised by loss of the epithelial marker E-cadherin and apico-basal polarity, expression of mesenchymal genes, acquisition of enhanced intrinsic migratory and invasive capabilities, and a reduced proliferation rate. These features are clinically relevant prognostic factors, and have been shown to correlate with increased metastasis and drug resistance^7^,^8^.

Multiple lines of evidence suggest that the tumour microenvironment plays a key role in the induction of EMT, as reviewed in Talbot *et al*.^9^. In relation to hypoxia specifically, it is known that hypoxia promotes a series of gene expression changes (affecting approximately 1–1.5% of the genome), mediated by the stabilization of the hypoxia-inducible transcription factor HIF-1α^10^. Further strengthening the association between hypoxia and EMT, a significant number of the genes, regulated by hypoxia and HIF-1α, are also involved in metastasis^11^. Furthermore, the E-cadherin-repressing transcription factor TWIST is positively regulated by HIF-1α^12^. Moreover, Notch signalling enhances EMT, under hypoxic conditions, through the activation of the transcription factor SNAIL^13^. In these experiments, tumour cell populations were exposed to hypoxic conditions and profiled for changes in EMT-related properties. Within 72 hours of the exposure of epithelial carcinoma cells to hypoxia, the cells had undergone a profound morphological change to a fibroblastoid phenotype, consistent with the occurrence of EMT, and exhibited the hallmarks of EMT including SNAIL expression, β-catenin nuclear localisation, downregulation of E-cadherin, and increased migration potential and invasiveness^14^.

The cancer stem cell (CSC) hypothesis postulates the existence of a subpopulation of cancer cells with a high capacity for self-renewal and the ability to regenerate the heterogeneity of the parental tumour, compared to differentiated non-CSC counterparts^15^. Conversely, recent studies have shown that plasticity is also an intrinsic property of differentiated cancer cells, whereby CD44^lo^CD24^hi^ non-CSCs can spontaneously dedifferentiate into CD44^hi^CD24^lo^ CSCs under certain conditions^16^,^17^. Indeed, it has been hypothesised that the dynamic interconversions between non-CSCs and CSCs can significantly increase the tumorigenic and metastatic potential of human breast cancers^17^. A metastatic growth is hypothesised to require the presence of at least one CSC^2^ that has reached the distant site by virtue of its EMT/mesenchymal properties. However, metastatic lesions typically display an epithelial morphology similar to that of the primary tumour. This apparent paradox may be explained by the transient nature of EMT and the existence of the reverse process, termed the mesenchymal-epithelial transition (MET), which is activated by signals emanating from the microenvironment of the distant site. MET restores the proliferative capacity of tumor cells, deposited at a distant site, thus driving the outgrowth of micro-metastatic lesions^18^,^19^,^20^,^21^. These dynamic EMT/MET interconversions further underscore the importance of cellular plasticity in tumour progression.

Dedifferentiation of differentiated cells has been reported to occur in hypoxic regions at higher frequencies compared to normoxic regions within the same tumours^2^. Experimentally, it has also been shown that hypoxic conditions promote dedifferentiation in ductal breast carcinoma *in situ* (characterised by increased nuclear to cytoplasmic ratio, loss of polarity, downregulation of ERα and upregulation of the epithelial breast stem cell marker CK19), suggesting the acquisition of cellular attributes reminiscent of a “stem-like” state^22^. Consistent with these observations, it has been reported that highly tumourigenic fractions of cells are preferentially located within the hypoxic regions of neuroblastomas^23^. Furthermore, upregulation of the CSC cell surface marker CD133 has also been reported in hypoxic regions of medulloblastomas^24^.

We recently identified the ganglioside GD2, a cell surface glycosphingolipid, as a breast CSC marker^25^. We also showed that GD2(+) cells express mesenchymal markers and exhibit increased mammosphere-forming efficiency and tumor-initiating capacity, relative to GD2(–) counterparts. Moreover, GD2(+) cells overlap with the CD44^hi^/CD24^lo^ subpopulation, known to be enriched for breast CSCs^25^. Also, GD3S—the enzyme which catalyses the formation of GD3, the immediate precursor of GD2—is essential for generating CSCs through EMT^26^. These findings further reinforce the links between the acquisition of CSC attributes and the occurrence of EMT.

The development of the phenotypic and functional heterogeneity, observed within tumours, is thought to be driven by selection pressures imposed by ever-changing microenvironmental conditions^27^. Experimentally, it has been observed that the events responsible for triggering these phenotypic changes are those that require significant metabolic adaptations for cells to survive, such as hypoxia and oxidative stress^28^. As alluded to previously, it has been hypothesised that hypoxia induces a stem cell phenotype in non-stem cancer cells^29^. It has also been demonstrated that prolonged exposure to hypoxia can result in a phenotypic shift in the non-stem population towards a population enriched for cancer cells with more aggressive properties, in terms of self-renewal capabilities and EMT features, in essence “reprogramming” of non-stem cells into a stem-like state^6^.

The clinical implications of hypoxia for cancer therapy are also of great importance, most evidently in the context of radiation and chemotherapy. It has been established that resistance to radiation is conferred by hypoxia, and the mechanism by which this is achieved is well understood^30^. Thus, radioresistance arises in the hypoxic regions because of the relatively fewer oxygen-derived free radicals, generated by ionising radiation, that compromise the cytotoxic effects of radiotherapy. In the case of chemotherapy, hypoxia has also been shown to elicit an overall decrease in the efficacy of chemotherapeutic agents *in vivo*^3^, but the underlying mechanisms are less understood.

In this work, we studied the effects of hypoxia on Ras-transformed human mammary epithelial (HMLER) cells via *in vitro* experiments and *in silico* mathematical modelling. The effects of hypoxia were analysed through five primary *in vitro* methods, including cell morphology, metabolism- and EMT-related gene expression, GD2-mediated fractionation of CSCs, cell growth, and mammosphere assays as an indicator of CSC self-renewal/plasticity. We also show that the experimental results can be predicted theoretically through ordinary differential equation-based mathematical modelling and stochastic simulations.

## Methods

### Statistical analysis of gene expression signature overlap

The methods used in this work for the analysis of the obtained gene expression signatures were as described in Zarkoob *et al*.^31^. Gene signatures were obtained by comparing the observed normalized signal intensity, for each probe tested on the microarray, to the observed normalized signal intensity of the normal cell population. Probes, which showed a signal two or more times higher than the normal cell population, were taken as the upregulated gene signature for that population of cells. Likewise, for those probes, which showed a signal two or more times lower than the normal cell population, were taken as the signature of downregulated genes. In determining the statistical significance of whether two sets of probe signatures overlapped, the lists were first compared via a confusion matrix, which shows the degree of the overlap between the lists, and the complement of the lists (i.e. all probes which were measured, but exclusively in one list or another, or in neither). From this confusion matrix, by the Fisher Two-Sided Exact Test, a p-value for the degree of statistical significance of overlap was determined.

### Mathematical modelling

**Figure 1:**
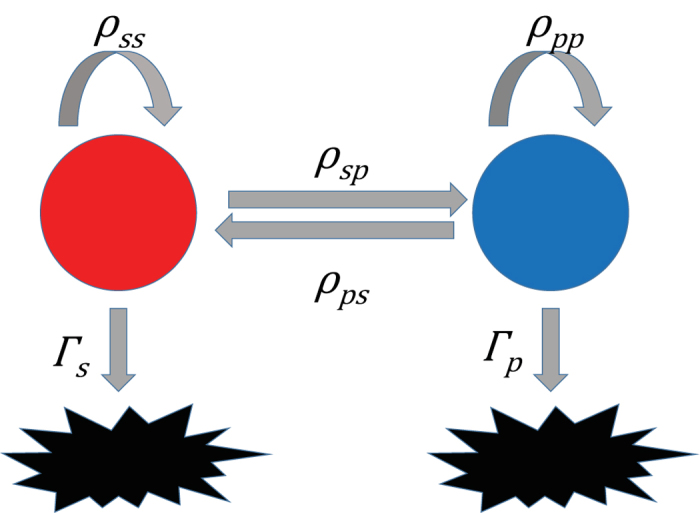
Schematic representation of the two compartment model. Red indicates CSC-like cells, and blue indicates non CSC-like cells. These cells may transition between either type, either type may self-replicate, or may fail to replicate/undergo apoptosis (black figures). The parameters, describing each of these first order transition rates, are as indicated in the figure.

We applied a two-compartment model (Fig. 1), consisting of two phenotypically distinct cellular subpopulations of biomarker-positive and -negative cells—namely CSCs and non-CSCs—each with the ability to self-replicate, die, and the ability to transition into the other phenotype. A brief description of the parameters of the model is presented in Fig. 1. The details of the mathematical model are presented in the Supplemental Information. The deterministic behaviour for each of the subpopulations was modelled using a system of ordinary differential equations, and the stochastic behaviour is described by the corresponding Master equation. The equations were solved numerically, and stochastic simulations were performed employing the well-known Gillespie algorithm^32^. The model parameters were estimated using the experimental data for the fraction of CSCs (GD2+) and the mammosphere-formation efficiencies in both normoxic and hypoxic conditions.

Parameters were fit using a computational brute-force search through the parameter space over all possible combinations of parameters, within a fixed gridspace. The parameter sets which provided the closest solutions to the model within a given tolerance were taken as possible parameter sets. Those parameters, presented within this work, represent the average of the given parameter over all such sets. For completeness, we also present the standard deviation of these values over the determined space of solutions. The model was able to fit well to the experimental data, and captured the growth of cancer cells at both low and high cell number limits as well as the dynamics of cell growth.

A stochastic implementation of the mathematical model, using the cellular reactions described in Fig. 1 (i.e. the two-compartment model), was simulated via the Gillespie algorithm. Using this with the initial condition of negative biomarker cells, the model was simulated over 100 runs. For each of these runs, if the total number of cells at the simulation end time exceeded 20, the run was considered positive for a mammosphere. The total proportion of mammospheres formed in this way represents the *in silico* mammosphere formation rate, as simulated by the stochastic model.

### Cell culture

HMLER cells were grown as previously described^5^ and under hypoxic conditions (1% O_2_; 5% CO_2_) using a hypoxic chamber (courtesy of the Gordon Mills lab, MD Anderson Cancer Centre, Houston, TX, USA). Mammosphere formation was assayed by seeding 3000 cells (per well) of 96-well ultra-low attachment plates (Costar) in mammosphere-formation media^33^. Fresh medium was added every two days. Mammospheres, greater than 60 micrometres, were counted following 10 days of culture. FACS-sorting of cells, according to GD2 expression, was performed as previously described^25^.

### Microarrays

Three hundred nanograms of Total RNA were amplified and purified using Illumina TotalPrep RNA Amplification Kit (Ambion, Cat# IL1791) according to the manufacturer’s instructions. Briefly, first strand cDNA was synthesized by incubating RNA with T7 oligo(dT) primer and reverse transcriptase mix at 42 °C for 2 hours. RNase H and DNA polymerase master mix were immediately added to the reaction mixture following reverse transcription, and were incubated for 2 hours at 16 °C to synthesize second strand cDNA. RNA, primers, enzymes and salts that would inhibit *in vitro* transcription were removed through cDNA filter cartridges (part of the amplification kit). *In vitro* transcription was performed and biotinylated cRNA was synthesized by 14-hour amplification with dNTP mix containing biotin-dUTP and T7 RNA polymerase. Amplified cRNA was subsequently purified and the concentration measured by a NanoDrop ND-1000 Spectrophotometer (NanoDrop Technologies, DE). An aliquot of 750 nanograms of amplified products was loaded onto Illumina Sentrix Beadchip Array hum HT-12.v4 arrays, hybridized at 58 °C in an Illumina Hybridization Oven (Illumina, Cat# 198361) for 17 hours, washed and incubated with streptavidin-Cy3 to detect biotin-labelled cRNA on the arrays. Arrays were dried and scanned with BeadArray Reader (Illumina, CA). Data were analysed using GenomeStudio software (Illumina, CA). Clustering and pathway analysis were performed with GenomeStudio and Ingenuity Pathway Analysis (Ingenuity Systems, Inc.) software respectively.

## Results

**Figure 2:**
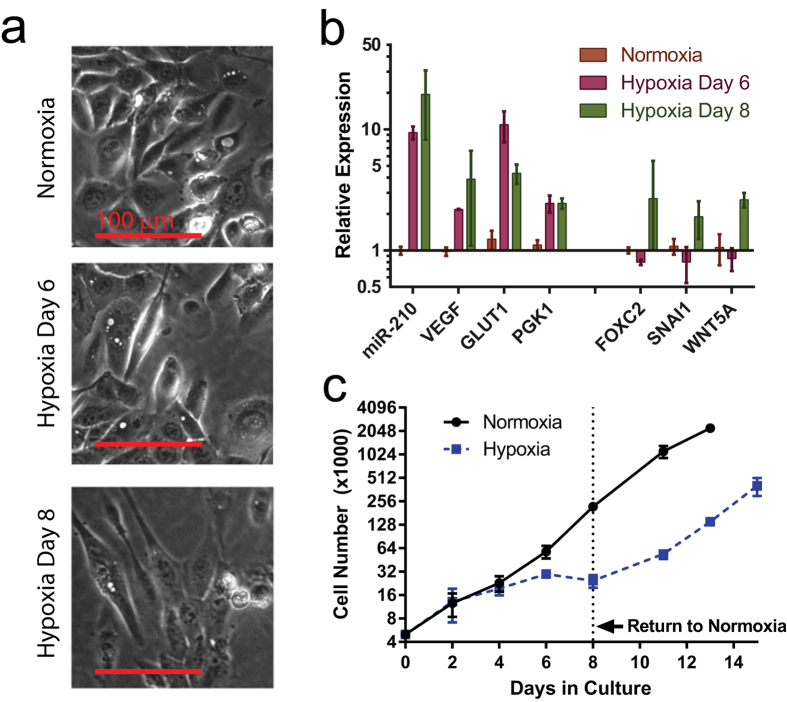
Prolonged exposure of HMLER cells to hypoxia induces a mesenchymal morphology and changes in gene expression. (**a**) Representative images of HMLER cells grown in normoxic conditions or in hypoxic (1% O_2_) conditions for 6 or 8 days. (**b**) RT-PCR for the indicated genes was performed using RNA extracted from HMLER cells exposed to the indicated conditions. Gene expression was measured relative to GAPDH and normalized to normoxia. (**c**) HMLER cell growth rates were monitored by counting live cells, based on trypan blue exclusion, at the indicated timepoints during exposure to normoxia or hypoxia. At 8 days, the hypoxia-treated cells were transferred to normoxic conditions.

In order to profile hypoxia-induced changes in EMT-related gene expression and CSC properties, we cultured Ras-transformed immortalized human mammary epithelial cells (HMLER) under normoxic (20% O_2_, 5% CO_2_) (hereafter termed HR-N) or hypoxic (1% O_2_, 5% CO_2_) conditions. Initially, we sought to establish a timecourse of morphological and gene expression changes, related to EMT induction following exposure to hypoxia. While we expected to observe a rapid transition to a mesenchymal phenotype within 24 hours^12^, we observed instead that exposure to hypoxia for up to six days (hereafter termed HR-H6) was not sufficient to alter the ‘cobblestone-like’ epithelial growth pattern of the rounded HMLER cells to the elongated, spindle-shaped morphology associated with mesenchymal cells and cells that have undergone EMT. Somewhat remarkably, an additional 2 days of culture under hypoxic conditions—i.e. following a total of 8 days of exposure to hypoxia (hereafter termed HR-H8)—was sufficient to promote the acquisition of an elongated, spindle-like cellular morphology (Fig. 2A), consistent with the phenotypic conversion of epithelial HMLER cells to mesenchymal counterparts via the process of EMT. Interestingly, analysis of the gene expression profiles of HMLER cells at these distinct timepoints, following exposure to hypoxia (HR-H6 and HR-H8), corroborates the profound differences seen in cell morphology between these 2 timepoints (HR-H6 vs Hr-H8). While 6 days of hypoxic exposure (HR-H6) was sufficient to elicit expression of the known hypoxia-induced genes miR-210, VEGF, GLUT1, and PGK1^34^,^35^, the expression of known EMT-associated genes FOXC2, SNAI1 and WNT5A^36^ was not induced until the day 8 timepoint (HR-H8; Fig. 2B). Notably, the cell growth rate of HMLER cells, cultured under hypoxic conditions, is also strikingly different between 6 and 8 days of hypoxic exposure. Despite an initial overlap in the cell growth rates between normoxic and hypoxic conditions (days 0–4), by day 6 (HR-H6) the growth rate was suppressed compared to normoxic counterparts (HR-N), and by day 8 (HR-H8), there was a pronounced decrease in the total number of viable cells. Based on these findings, we chose to investigate how prolonged exposure of HMLER cells to hypoxic growth conditions alters EMT and CSC properties and, in particular, we aimed to understand the differences between HMLER cells exposed to hypoxia for 6 days (HR-H6) and 8 days (HR-H8).

### Hypoxia-induced global gene expression

**Figure 3:**
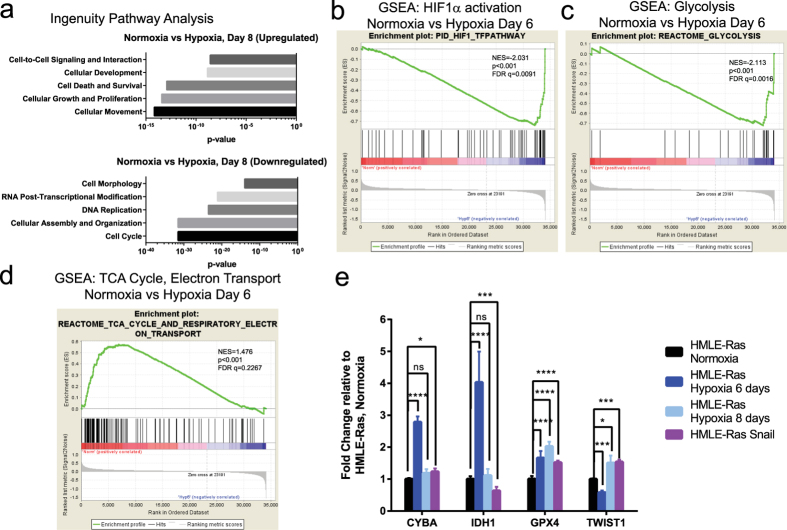
Analysis of the global changes in gene expression caused by hypoxia confirms induction of a broad cellular response. (**a**) Ingenuity Pathway Analysis of the microarray results comparing normoxia- and hypoxia-treated cells demonstrates a significant enrichment for cell biological categories associated with EMT (cell movement and cell morphology) or with proliferation (cell growth and proliferation and cell cycle). (**B**–**D**) GSEA analysis of the microarray results comparing normoxia- and hypoxia-treated cells was performed using the c2cp curated set of canonical pathways. The results demonstrate a significant enrichement for higher expression of known HIF1α target genes in hypoxia treated- cells (indicated by a greater density of black vertical lines on the right side of the graph). (**b**), higher expression of glycolysis-associated genes in hypoxia treated- cells (similarily indicated) (**c**), and lower expression of respiratory electron transport genes in hypoxia treated- cells (indicated by a greater density of black vertical lines on the left side of the graph) (**d**), compared to normoxic counterparts. (**e**) Graph depicting the fold changes in gene expression, as recorded by microarray analysis for the indicated probes, comparing HMLER cells grown under normoxic conditions and under hypoxic conditions after 6 days, as well as HR-SN cells which express the EMT-inducing transcription factor Snail, *p < 0.05, **p < 0.01, ***p < 0.001, ****p < 0.0001.

In order to assess the impact of hypoxic exposure on global gene expression, we extracted RNA and performed microarray gene expression analysis. In addition to HR-N, HR-H6 and HR-H8 cells, we also measured gene expression from HMLER cells transduced with a SNAI1-expression vector encoding the EMT-activator Snail (HR-SN). The two-sided Fisher exact test was performed on gene signatures to determine the degree of statistical significance of overlapping genes present in multiple signatures, as described in Zarkoob *et al*. (2013) and the Methods section. Notably, a number of comparisons did show statistical significance. As expected, it was shown that the upregulated genes in HR-H6 and HR-H8 showed an overlap that was statistically significant, and considerably more significant than could be due to chance alone (Fig. 3A).

In concordance with our findings above, whereby HR-H6 cells did not express EMT-associated genes, the degree of overlap between the upregulated gene signatures for HR-H6 and HR-SN did not meet the criteria for statistical significance. However, the analogous comparison between HR-H8 and HR-SN cells was indeed statistically significant (p < 0.05, Two-Sided Fisher Exact Test), suggesting that there is a greater degree of similarity between the upregulated gene signatures of HR-H8 cells and HR-SN cells that is not apparent at the earlier timepoint (HR-H6). Collectively, these results indicate that the genes upregulated during the hypoxic response of HMLER cells, at 6 days post-hypoxia induction (HR-H6), are, in fact, distinct from the genes underpinning the established mesenchymal phenotype of the HR-SN cells. Given that the upregulated genes of HR-H8 cells show significant overlap with those of HR-SN cells, this, in turn, suggests that the dynamic process of hypoxia-induced EMT unfolds between days 6 and 8 post-hypoxia exposure in the HMLER model. Moreover, our data indicate that the hypoxic response *per se* is initiated before day 6 and precedes the activation of the EMT program and the establishment of the hallmarks of EMT. Additionally, it should be noted that when comparing the gene signatures for downregulated genes between HR-H6 and HR-SN and also between HR-H8 and HR-SN, both comparisons were statistically significant (Fig. 3A).

Of note, other effects of hypoxia on gene expression, observed in this model, represent differences in genes known to be associated with therapeutic response. Indeed, it is known that microenvironmental factors affect the efficacy of conventional therapeutic regimens, and that cells that have undergone EMT and CSCs are resistant to standard therapies such as radiation and chemotherapy. The downregulation of the gene *BRCA1* (breast cancer 1, early onset – exon 4, transcriptional variants 1b, delta 11b, delta 14–17) has been shown to correlate with an insensitivity to the chemotherapeutic agent doxorubicin^37^. As indicated in Supplemental Table S1, *BRCA1* expression was downregulated following exposure to hypoxia and Snail-induced EMT. Additionally, we noted the upregulation of Twist1, in HR-H8 and HR-SN cells compared to HR-N cells, which has been shown to increase the resistance to microtubule-disrupting anticancer drugs, such as taxanes^38^. Furthermore, in all hypoxic cell populations, we observed increased levels of *MUC1* (Mucin 1), a gene encoding an oncoprotein that is aberrantly overexpressed in human breast carcinoma and blocks irradiation- and hypoxia-induced cell death^39^,^40^. Baumann *et al*.^41^ have shown that the inhibition of EGFR variants may correlate with radioresistance, and, accordingly, we observed downregulation of EGFR variants in hypoxic cells after 8 days. The fold changes in expression of each of the aforementioned genes (relative to HR-N cells) are presented in Supplemental Information (Supplemental Table S1). Taken together, these gene expression changes point to potential molecular avenues by which both radioresistance and chemoresistance may occur in a hypoxia-exposed populations of cells (and correlate with similar changes in HR-SN cells).

### Gene ontology analyses

The hypoxia-derived gene signatures were profiled for association with existing datasets through Ingenuity Pathway Analysis (QIAGEN), gene ontology classification (DAVID)^42^ and gene set enrichment analysis (GSEA)^43^. The ingenuity pathway analysis revealed that the most enriched pathways in hypoxic HMLER cells after 8 days (HR-H8) were highly important for cancer (e.g. Cellular Growth and Proliferation, Cell Death and Survival) and for EMT (Cellular Movement, Cell Morphology) (Fig. 3B). These analyses suggest that the molecular mechanisms underpinning the changes, observed within the gene expression signatures of the cells exposed to hypoxia for a prolonged period of time (HR-H8), are likely due to an interaction between the aforementioned molecular pathways, resulting in gene activation or silencing and an altered phenotype as well as novel behavioural attributes.

We next used GSEA to identify functional pathways and datasets altered by hypoxic exposure (Fig. 3B–D) by comparing the changes in gene expression, uncovered in our microarray analysis, to the curated gene sets for canonical pathways (c2cp) maintained by the Broad Institute^43^; gene lists are presented in Supplementary Fig. S1. Importantly, we observed increased expression of HIF1α target genes in HR-H6 cells when compared to HR-N cells (Fig. 3B). Strikingly, there is also a shift from the expression of genes associated with the tricarboxylic acid cycle (TCA) and the electron transport chain (also known as the respiratory chain) in normoxia-treated cells towards an enrichment of genes encoding glycolytic enzymes in hypoxia-treated cells, a well-established association (Fig. 3C,D)^44^,^45^. These data suggest that hypoxic HMLER cells undergo metabolic reprogramming to suppress engagement of the TCA cycle and oxidative phosphorylation/electron transport, and exhibit a shift towards anaerobic glycolysis, for the generation of ATP, to meet their energy needs and sustain a pro-growth metabolic program.

Additionally, our microarray analyses support an increase in CSC-associated genes following exposure to hypoxia. First, we determined that TWIST1, a gene known for promoting a CSC phenotype in breast cancer^12^,^46^, is upregulated in HR-H8 cells, as compared to HR-N and HR-H6 cells (Fig. 3E). This suggests that the upregulation of Twist expression, which is implicated in a plastic transition to a more stem-like state, occurs after prolonged exposure to hypoxia and is not necessarily in the initial set of upregulated genes. This finding leads us to predict an increase in the proportion of CSCs in the hypoxic microenvironment from day 6 to day 8. Additionally, CYBA (cytochrome b-245, alpha polypeptide), GPX4 (Glutathione peroxidase 4), and IDH1 (Isocitrate dehydrogenase 1) are genes, which, in prior studies, have been found to be elevated in breast CSC-enriched populations^47^, and are involved in the metabolism of reactive oxygen species (ROS). Consistent with these findings, our results show the upregulation of the said genes under hypoxic conditions (Fig. 3E). In a physiological setting, this finding is consistent with the notion that hypoxia predisposes to reperfusion injury, via the generation of ROS, oxidative stress and inflammation as has been shown for breast cancers^48^. While, our data is derived from *in vitro* experimentation, we nevertheless observed that CYBA and IDH1 increase transiently in HR-H6 cells but decrease to a slightly elevated level above normoxia in HR-H8 cells, while GPX4 increases continually, up to 8 days of hypoxia. Moreover, we note that these genes are not significantly differentially expressed in HR-SN cells, as compared to control counterparts, again suggesting that changes in ROS metabolism are changes conferred uniquely by hypoxia, not manifested in non-hypoxia/Snail-induced EMT.

### Hypoxia increases CSC properties

**Figure 4:**
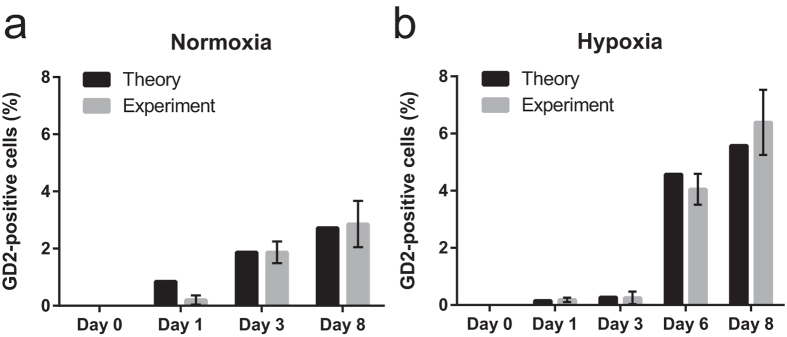
Generation of GD2(+) cells from FACS-sorted GD2(−) HMLER cells, following exposure to normoxia or hypoxia, and comparison of experimental data with theoretical predictions. (**a**) Proportion of GD2(+) HMLER cells which emerge spontaneously from GD2(−) HMLER cells, with extended time in culture under normoxic conditions. (**b**) Proportion of GD2(+) HMLER cells, generated from GD2(−) HMLER cells, during exposure to hypoxia in culture.

We recently described the ganglioside GD2 as a novel breast CSC-specific cell surface marker^25^. In order to determine the influence of oxygen levels in the microenvironment on the percentage of GD2(+) cells, we FACS-isolated a pure GD2(−) population, and subsequently assayed for the conversion of GD2(−) to GD2(+) cells under normoxic and hypoxic conditions. For the purposes of modelling and interpretation of the experimental results, the initial value for the number of GD2(+) cells in culture is taken as zero. The experimental results show that the proportion of GD2(+) cells grows to an equilibrium value, which is a small but non-zero value, and the time required to reach this steady state is more than 8 days under normoxic conditions (Fig. 4A).

In comparison, the change in the proportion of GD2(+) cells, as a function of time, was also obtained under hypoxic conditions (1% O_2_), as illustrated in Fig. 4B. In this case, the proportion of GD2(+) cells increases up to an equilibrium value larger than that of the normoxic cells. Under normoxic conditions, the fraction of GD2(+) cells increases towards the steady state value, over the entire time period studied (representative data are presented in Supplementary Fig. S2). In contrast, under hypoxic conditions, the cells modify their behaviour between days 3 and 6, such that, within this time interval, there is an increase in the proportion of GD2(+) cells, not seen before day 3 (Fig. 4B). This is consistent with the notion that during exposure to hypoxic conditions, after a certain critical timepoint, there exists a deterministic switch that enhances the plasticity of HMLER cells, preceding dedifferentiation and EMT.

**Figure 5:**
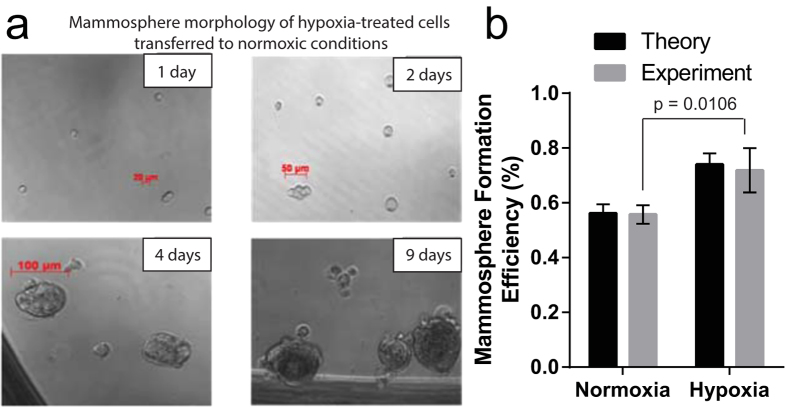
Effects of normoxic and hypoxic conditions on the mammosphere-forming efficiency of HMLER cells. (**a**) Representative images of mammospheres, formed by HMLER cells exposed to hypoxia for 8 days, and subsequently plated for mammosphere formation under normoxic conditions, at 1, 2, 4, and 9 days after plating. (**b**) Mammosphere-forming efficiency of HMLER cells exposed to normoxia or hypoxia and subsequently subjected to a mammosphere formation assay under normoxia.

A second assay, used to estimate the stem cell frequency in a population, measures the proportion of cells that form spheres under low-attachment cell culture conditions^33^. However, in this experiment, hypoxia drastically reduced cell growth (Fig. 2C), confounding the measurement of the expected increase in mammosphere formation due to the increased proportion of GD2(+) cells, caused by exposure to hypoxia. In order to circumvent this limitation, we returned hypoxia-treated 2D-cultured cells to a normoxic environment for the duration of the mammosphere formation assay. Under these conditions, hypoxia-treated cells formed mammospheres (Fig. 5A) at a statistically significantly greater extent than cells continuously maintained in normoxic conditions, consistent with the increased proportion of GD2(+) cells in hypoxia-treated populations (Fig. 5B).

### Mathematical modelling indicates a hypoxia-induced increase in plasticity

**Figure 6:**
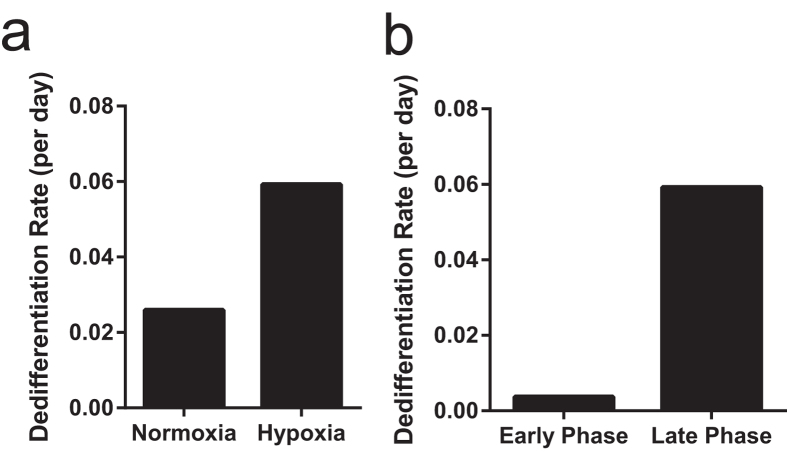
Computational modelling of HMLER cell dedifferentiation following exposure to normoxia or hypoxia. (**a**) Dedifferentiation rates of HMLER cells under normoxic and hypoxic conditions, based on our computational model. (**b**) Dedifferentiation rates, as described by parameter sets obtained for experimental data, fit to both early (before day 3) and late (after day 3) time intervals, following exposure to hypoxia, using the computational model.

A mathematical model was used to compare cancer cell heterogeneity and plasticity under both normoxic and hypoxic microenvironmental conditions. The model parameters were extracted by comparing the modelling results for the fraction of CSCs (using deterministic equations) and mammosphere formation analysis (using stochastic simulations) to the corresponding experimental data, as described in the Methods section. This comparison reveals that the only parameter, which significantly varies when the microenvironment changes from normoxia to hypoxia, is the rate of dedifferentiation, i.e. the rate of conversion of non-CSCs to CSCs, which is representative of the observed increase in cell plasticity following exposure to hypoxia (Fig. 6). It appears that the degree of plasticity increases by decreasing the concentration of oxygen (i.e. by inducing a hypoxic microenvironment). However, the acquisition of plasticity does not proceed in a uniform manner. In fact, for the hypoxic conditions, the best fits to the mathematical model were obtained by assuming that the dedifferentiation rate is different for early and late time intervals (approximately, before and after day 3). This means that exposure to hypoxia initially decreases tumour cell plasticity (compare recovery of GD2(+) cells in normoxia vs hypoxia within 3 days), but significantly increases it at the later timepoints. As shown in Fig. 6, the dedifferentiation rate is approximately ten-fold greater at the later time (after day 3), compared to the corresponding value at the earlier time (before day 3).

## Discussion

In this study, we used a combination of CSC assays, gene expression analyses, and mathematical modelling to study the phenotypic plasticity underlying the conversion from a differentiated to a stem-cell phenotype under both normoxic and hypoxic conditions.

A primary result of this work describes the adaptation of transformed human mammary epithelial cells to growth under hypoxic conditions. Thus, we showed that hypoxic cells undergo a biphasic adaptation involving both gene expression changes as well as phenotypic alterations. Unlike HR-H8 cells exposed to hypoxia for 8 days, HR-H6 cells did not show as strong a statistical correlation with cells that have undergone a Snail-driven EMT. This suggests the occurrence of important changes, between days 6 and 8 of the hypoxia timecourse, which culminate in the acquisition of an EMT-like phenotype. More specifically, for those genes that must be downregulated to permit the cell to undergo an EMT-like transition, the repression occurs largely within the first 6 days, via a similar mechanism as within HR-SN cells. However, the analogous process for the genes which must be upregulated to reach an EMT-like state is different in the sense that the induced hypoxic response may involve a separate or delayed pathway of gene activation to reach a mesenchymal state. By the eighth day of exposure to hypoxia, the upregulated gene expression signature of HR-H8 cells more strongly matches the upregulated gene signature of HR-SN cells, suggesting that, at this point, a more complete EMT-like process is underway.

To further analyse the differences between the mechanisms governing these phenotypic transitions in response to a hypoxia timecourse, gene set functional analyses were carried out. These showed that, as expected, the primary drivers of the hypoxic phenotype were the HIF1α-related transcription factors and TNF1α. Moreover, GSEA reveals a switch from expression of TCA cycle genes to the expression of genes involved in the glycolytic pathway, in hypoxic cells at day 6, suggesting that this metabolic reprogramming occurs within 6 days during the transition from a normoxic to a stable hypoxic phenotype.

A second aspect of this study analysed the change in the cell surface expression of GD2, a glycosphingolipid and purported CSC biomarker, in HMLER cells exposed to hypoxia. Thus, we profiled the temporal dynamics of the transition from a GD2(−) population to a GD2(+) population, presumably comprised of a higher proportion of CSCs under the hypoxic regime. Moreover, the mammosphere formation assay was used to quantify the proportion of CSCs via a direct assay of their self-renewal and growth under ultra-low attachment conditions. The corresponding results must be interpreted accounting for stochastic variation in behaviour due to the small numbers of mammosphere-forming cells involved (HMLER cells have a lower ability to generate mammospheres compared to e.g. HMLER-Twist or HMLER-Snail cells). These two experiments, with results reconciled through mathematical modelling accounting for both deterministic and stochastic observed behaviour in a two-compartment model, show that the transition from normoxia to hypoxia induces a definite switch in the proportion of CSCs, with a higher proportion of CSCs emerging after hypoxia. Moreover, a quantitative parametric analysis of the mathematical model reveals that the nature of this differing phenotype between normoxia and hypoxia is due to a higher rate of dedifferentiation of non-CSCs to CSCs.

Importantly, this study highlights the fact that the phenotype of a tumour, in terms of the proportions of non-CSC/CSC (or differentiated vs. dedifferentiated) subtypes of cells comprising the lesion, will depend significantly on the microenvironmental parameters in the immediate vicinity of the tumour cells and the variability of these parameters within the tumour as a whole (e.g. tumour core vs tumour margin). That is, we have shown, for the studied cell line in particular, that oxygen conditions significantly impact gene expression and consequently modify the degree of cellular plasticity. For instance, model fitting during the induction of hypoxia necessitated the use of distinct parameter sets for early and late timepoints of exposure to hypoxia, representing a deterministic shift in behaviour. Thus, as an addendum to the result of Gupta *et al*.^49^, we note that the transition to a steady state tumour phenotype can only be viewed as a stochastic transition in a highly stable microenvironment, which is unlikely to occur in a growing *in vivo* lesion, where biological factors induce great microenvironmental variability.

## Additional Information

**How to cite this article**: Dhawan, A. *et al*. Mathematical modelling of phenotypic plasticity and conversion to a stem-cell state under hypoxia. *Sci. Rep*. **6**, 18074; doi: 10.1038/srep18074 (2016).

## Supplementary Material

Supplementary Information
