# Supplementary material for: Mathematical modelling of phenotypic plasticity and conversion to a stem-cell state under hypoxia
Source: Sci Rep. 2016 Feb 3;6:18074. doi: 10.1038/srep18074 (PMC4738268; doi:10.1038/srep18074)
Supplement: Supplementary Information [file srep18074-s1.docx]

**Supplemental Information**

**Mathematical modelling of phenotypic plasticity and conversion to a stem-cell state under hypoxia**

**Andrew Dhawan^1^*, Seyed Ali Madani Tonekaboni^2^*, Joseph H. Taube^3^*, Stephen Hu^3^, Nathalie Sphyris^3^, Sendurai A. Mani^3,4†^, Mohammad Kohandel^2,5†^**

**Mathematical model**

We assume a phenomenological two-compartment model for CSCs and non-CSCs which are separated based on the expression of the cell surface marker GD2. Moreover, we assume that the growth of each subpopulation is exponential. The following system of ordinary differential equations defines the deterministic behaviour of both CSCs (*S*), assumed to be positive for a CSC-biomarker, and non-CSCs (*P*), assumed to be CSC-biomarker negative:

$$\frac{dS}{dt}=\left( \rho_{ss}-\Gamma_{s}-\rho_{sp} \right)S+\rho_{ps}P$$

$$\frac{dP}{dt}=\left( \rho_{pp}-\Gamma_{p}-\rho_{ps} \right)P+\rho_{sp}S$$

Biological description of equation parameters:

| **Parameter** | **Description** |
| --- | --- |
| $\rho_{SS}$ | Self-renewal rate of CSCs |
| $\rho_{PP}$ | Self-renewal rate of non-CSCs |
| $\rho_{SP}$ | Transition rate from CSCs to non-CSCs |
| $\rho_{PS}$ | Transition rate from non-CSCs to CSCs |
| $\Gamma_{S}$ | Death rate of CSCs |
| $\Gamma_{P}$ | Death rate of non-CSCs |

In order to obtain the fraction of each subpopulation, we define:

$$F_{\mathrm{positive}}\left( t \right)=\frac{S\left( t \right)}{S\left( t \right)+P\left( t \right)}, F_{\mathrm{negative}}\left( t \right)=\frac{P\left( t \right)}{S\left( t \right)+P\left( t \right)}$$

In the steady state, we have:

$$F_{\mathrm{positive}}=\frac{\gamma_{1}+\sqrt{\gamma_{1}^{2}-4\gamma_{2}}}{2}, F_{\mathrm{negative}}=1-F_{\mathrm{positive}}, \gamma_{1}=\frac{{(\rho}_{pp}-\Gamma_{p})+\rho_{ps}+\rho_{sp}-(\rho_{ss}-\Gamma_{s})}{{(\rho}_{pp}-\Gamma_{p})-(\rho_{ss}-\Gamma_{s})}, \gamma_{2}=\frac{\rho_{ps}}{{(\rho}_{pp}-\Gamma_{p})-(\rho_{ss}-\Gamma_{s})}$$

In order to investigate the stochastic behaviour of the cancer cells in the two-compartment model, the Master equation for the joint probability can be written, and solved computationally. Stochastic simulations are performed using the Gillespie algorithm.

**Table of parameters:** The estimated parameters for normoxic and hypoxic conditions. ^*^Average value (standard deviation).

| Condition | $\boldsymbol{\rho}_{\boldsymbol{SS}}$ | $\boldsymbol{\rho}_{\boldsymbol{SP}}$ | $\boldsymbol{\Gamma}_{\boldsymbol{S}}$ | $\boldsymbol{\rho}_{\boldsymbol{PS}}$ | $\boldsymbol{\rho}_{\boldsymbol{PP}}$ | $\boldsymbol{\Gamma}_{\boldsymbol{P}}$ |
| --- | --- | --- | --- | --- | --- | --- |
| Normoxic | 0.329(0.07)^*^ | 0.416(0.04) | 0.245(0.06) | 0.026(1e-3) | 0.584(0.054) | 0.390(0.07) |
| Hypoxic (early time) | 0.369(0.09) | 0.372(0.05) | 0.259(0.09) | 0.004(1e-4) | 0.581(0.06) | 0.415(0.06) |
| Hypoxic (late time) | 0.369(0.09) | 0.372(0.04) | 0.259(0.09) | 0.059(1e-3) | 0.548(0.06) | 0.392(0.06) |

**Supplemental Table S1:** Fold-change of gene expression compared to normoxic baseline, as recorded by microarray, for key genes of interest among the cell types/treatments considered.

| **Gene Name** | **Normoxia**  **(HR-N)** | **Hypoxia Day 6**  **(HR-H6)** | **Hypoxia Day 8**  **(HR-H8)** | **Snail over- expression**  **(HR-SN)** |
| --- | --- | --- | --- | --- |
| XBP1 | 1 | 1.79 | 1.29 | 1.52 |
| MIR205 | 1 | 0.08 | 0.12 | 0.05 |
| ZEB2 | 1 | 1.05 | 0.97 | 1.55 |
| BRCA1-Exon 4 | 1 | 0.95 | 0.90 | 0.91 |
| BRCA1-transcriptional variant 1b | 1 | 0.87 | 0.92 | 0.97 |
| BRCA1-transcriptional variant delta 14-17 | 1 | 0.56 | 0.31 | 0.40 |
| BRCA1-transcriptional variant delta 11b | 1 | 0.52 | 0.31 | 0.37 |
| TWIST1 | 1 | 0.58 | 1.49 | 1.54 |
| TWIST2 | 1 | 0.91 | 0.91 | 0.94 |
| TWIST2-transcript variant 1 | 1 | 0.91 | 0.83 | 0.82 |
| TWIST2-transcript variant 2 | 1 | 0.63 | 0.66 | 1.34 |
| MUC1-transcript variant 1 | 1 | 1.93 | 1.23 | 1.02 |
| MUC1-transcript variant 5 | 1 | 11.59 | 4.03 | 1.23 |
| MUC1-transcript variant 6 | 1 | 16.90 | 6.07 | 1.69 |
| EGFR-transcript variant 1 | 1 | 1.41 | 2.62 | 0.53 |
| EGFR-transcript variant 2, probe 1 | 1 | 1.34 | 1.67 | 0.84 |
| EGFR-transcript variant 2, probe 2 | 1 | 1.21 | 1.09 | 0.90 |
| EGFR-transcript variant 4 | 1 | 1.14 | 0.96 | 0.86 |

**Supplemental Figure S1:** Gene sets used for GSEA analysis in Figure 3, derived from the canonical pathways set of gene sets in the curated portion of MSigDB.

PID_HIF1_TFPATHWAY

> HIF-1-alpha transcription factor network

ABCB1

ABCG2

ADM

AKT1

ALDOA

ARNT

BHLHE40

BHLHE41

BNIP3

CA9

CITED2

COPS5

CP

CREB1

CREBBP

CXCL12

CXCR4

EDN1

EGLN1

EGLN3

ENG

ENO1

EP300

EPO

ETS1

FECH

FOS

FURIN

GATA2

GCK

HDAC7

HIF1A

HK1

HK2

HMOX1

HNF4A

ID2

IGFBP1

ITGB2

JUN

LDHA

LEP

MCL1

NCOA1

NCOA2

NDRG1

NOS2

NPM1

NT5E

PFKFB3

PFKL

PGK1

PGM1

PKM2

PLIN2

RORA

SERPINE1

SLC2A1

SMAD3

SMAD4

SP1

TERT

TF

TFF3

TFRC

VEGFA

REACTOME_GLYCOLYSIS

> Genes involved in Glycolysis

ALDOA

ALDOB

ALDOC

ENO1

ENO2

ENO3

GAPDH

GAPDHS

GPI

LOC642969

PFKFB1

PFKFB2

PFKFB3

PFKFB4

PFKL

PFKM

PFKP

PGAM1

PGAM2

PGK1

PKLR

PKM2

PPP2CA

PPP2CB

PPP2R1A

PPP2R1B

PPP2R5D

TPI1

TPI1P1

REACTOME_TCA_CYCLE_AND_RESPIRATORY_ELECTRON_TRANSPORT

> Genes involved in The citric acid (TCA) cycle and respiratory electron transport

ACO2

ADHFE1

ATP5A1

ATP5B

ATP5C1

ATP5D

ATP5E

ATP5F1

ATP5G1

ATP5H

ATP5I

ATP5J

ATP5J2

ATP5L

ATP5O

ATP6

ATP8

BSG

COX1

COX2

COX3

COX4I1

COX5A

COX5B

COX6A1

COX6B1

COX6C

COX7A2L

COX7B

COX7C

COX8A

CS

CYC1

CYCS

CYTB

D2HGDH

DLAT

DLD

DLST

ETFA

ETFB

ETFDH

FH

IDH1

IDH2

IDH3A

IDH3B

IDH3G

L2HGDH

LDHA

LDHB

LOC283398

LOC642727

LOC646675

LOC646677

LOC650667

LOC650674

LOC650883

LOC651820

LOC727947

MDH2

MTND5P10

ND1

ND2

ND3

ND4

ND4L

ND5

ND6

NDUFA1

NDUFA10

NDUFA11

NDUFA12

NDUFA13

NDUFA2

NDUFA3

NDUFA4

NDUFA5

NDUFA6

NDUFA7

NDUFA8

NDUFA9

NDUFAB1

NDUFB1

NDUFB10

NDUFB2

NDUFB3

NDUFB4

NDUFB5

NDUFB6

NDUFB7

NDUFB8

NDUFB9

NDUFC1

NDUFC2

NDUFS1

NDUFS2

NDUFS3

NDUFS4

NDUFS5

NDUFS6

NDUFS7

NDUFS8

NDUFV1

NDUFV2

NDUFV3

NNT

OGDH

PDHA1

PDHB

PDHX

PDK1

PDK2

PDK3

PDK4

PDP1

PDP2

PDPR

SDHA

SDHB

SDHC

SDHD

SLC16A1

SLC16A3

SLC16A8

SUCLA2

SUCLA2P1

SUCLG1

SUCLG2

UCP1

UCP2

UCP3

UQCR11

UQCRB

UQCRBP1

UQCRC1

UQCRC2

UQCRFS1

UQCRH

UQCRHL

UQCRQ

**Supplemental Figure S2:** Complete FACS data for detection of GD2, at the indicated timepoints, and under the indicated conditions. HMLER cells were grown under normoxic conditions. At Day 0, HMLER cells were trypsinized, counted and labeled with anti-GD2 antibody (clone 2Q549 from abcam). Labeled cells were sorted to obtain a GD2-negative population (panel A). Sorted cells were then plated (5 x 10^5^ cells per 3.5 cm plate) and cultured under either normoxic or hypoxic (1% O_2_) conditions. Both normoxic and hypoxic cell cultures were passaged concurrently with flow cytometry. Following each passage 5 x 10^5^ cells were re-plated per 3.5 cm plate**.** Flow cytometry gating for cells (B) for singlets (C) and for identification of positive GD2 staining (D, E) was performed as indicated below.**
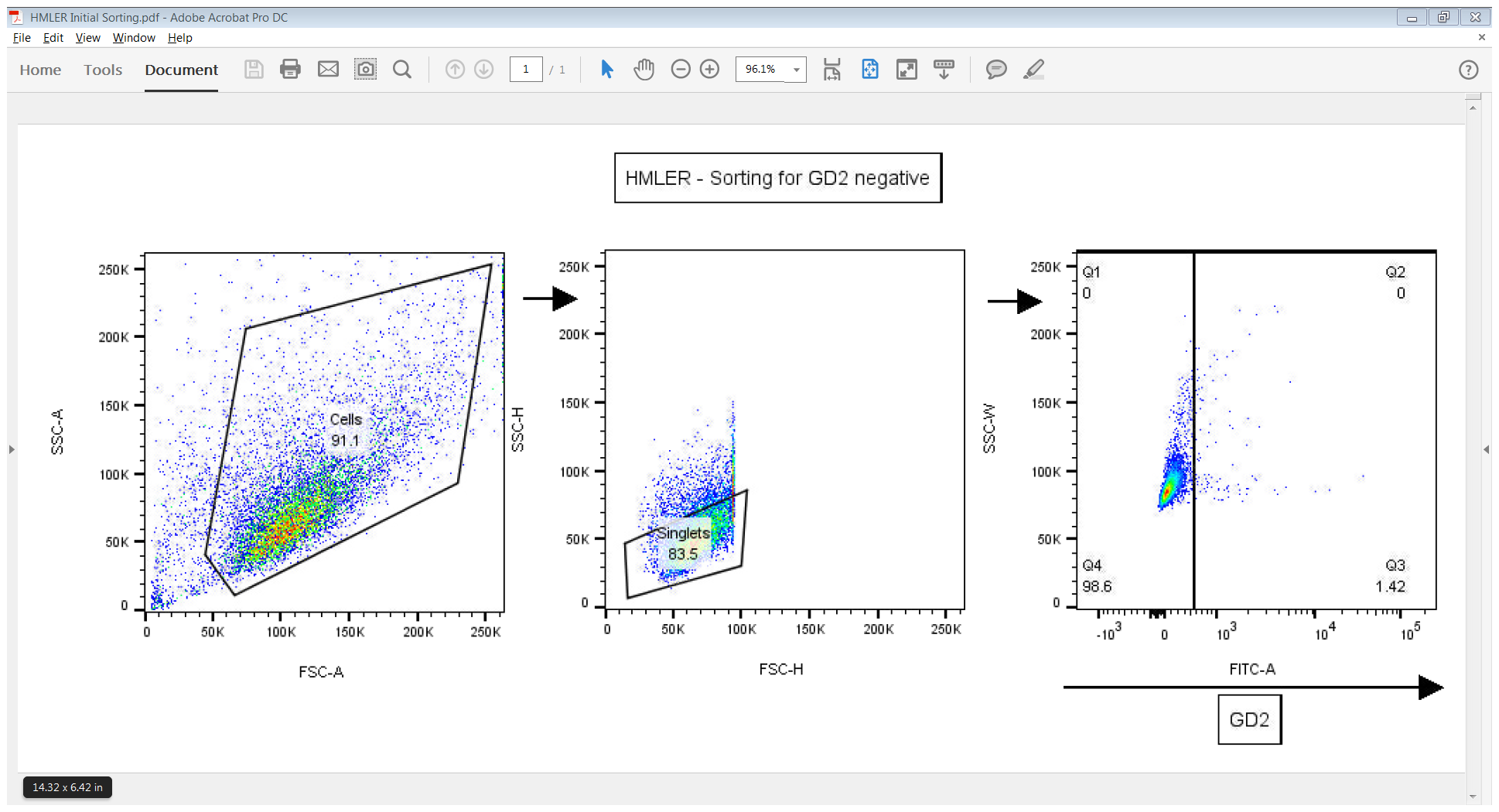
**

A


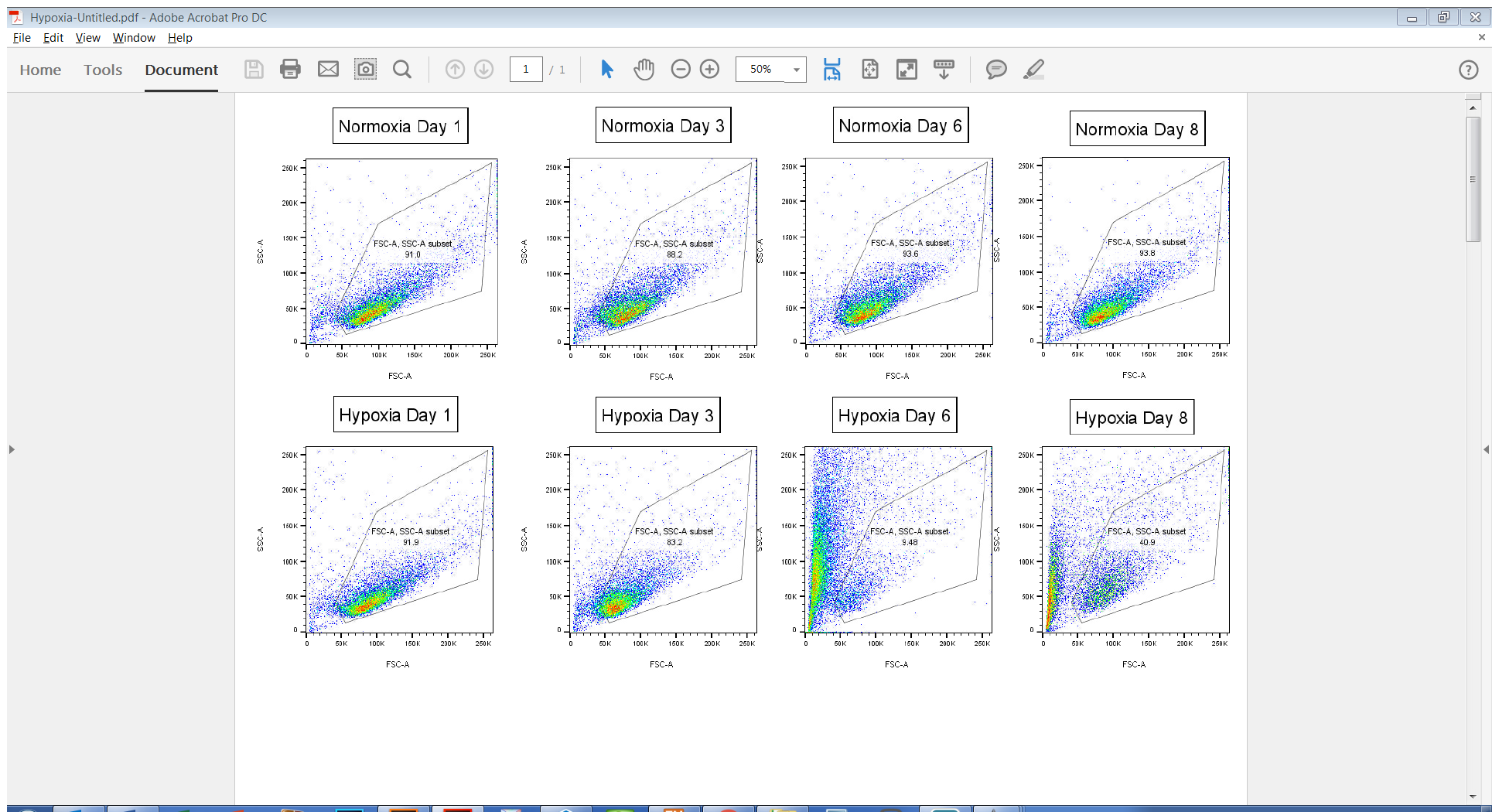


B


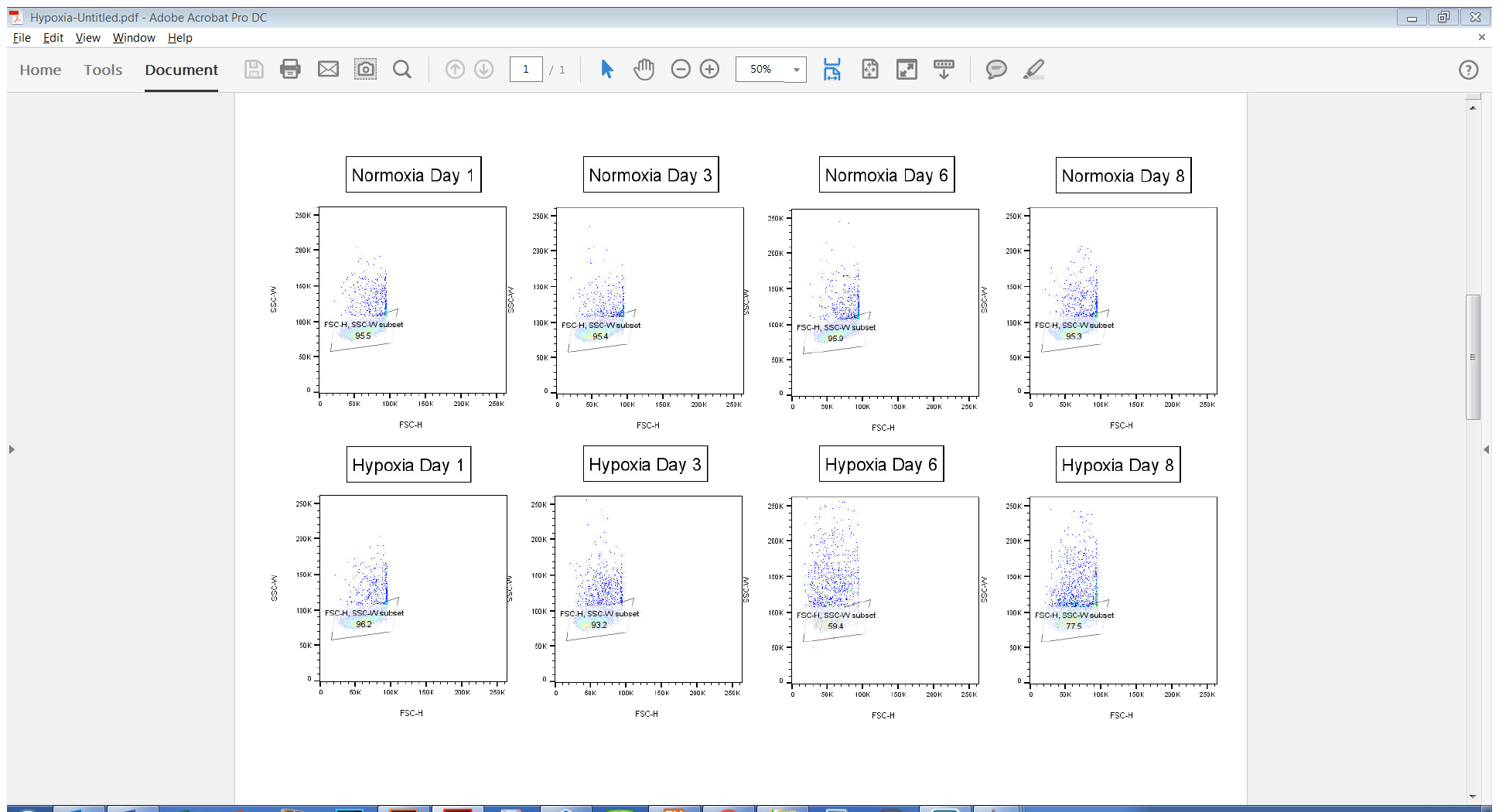


C


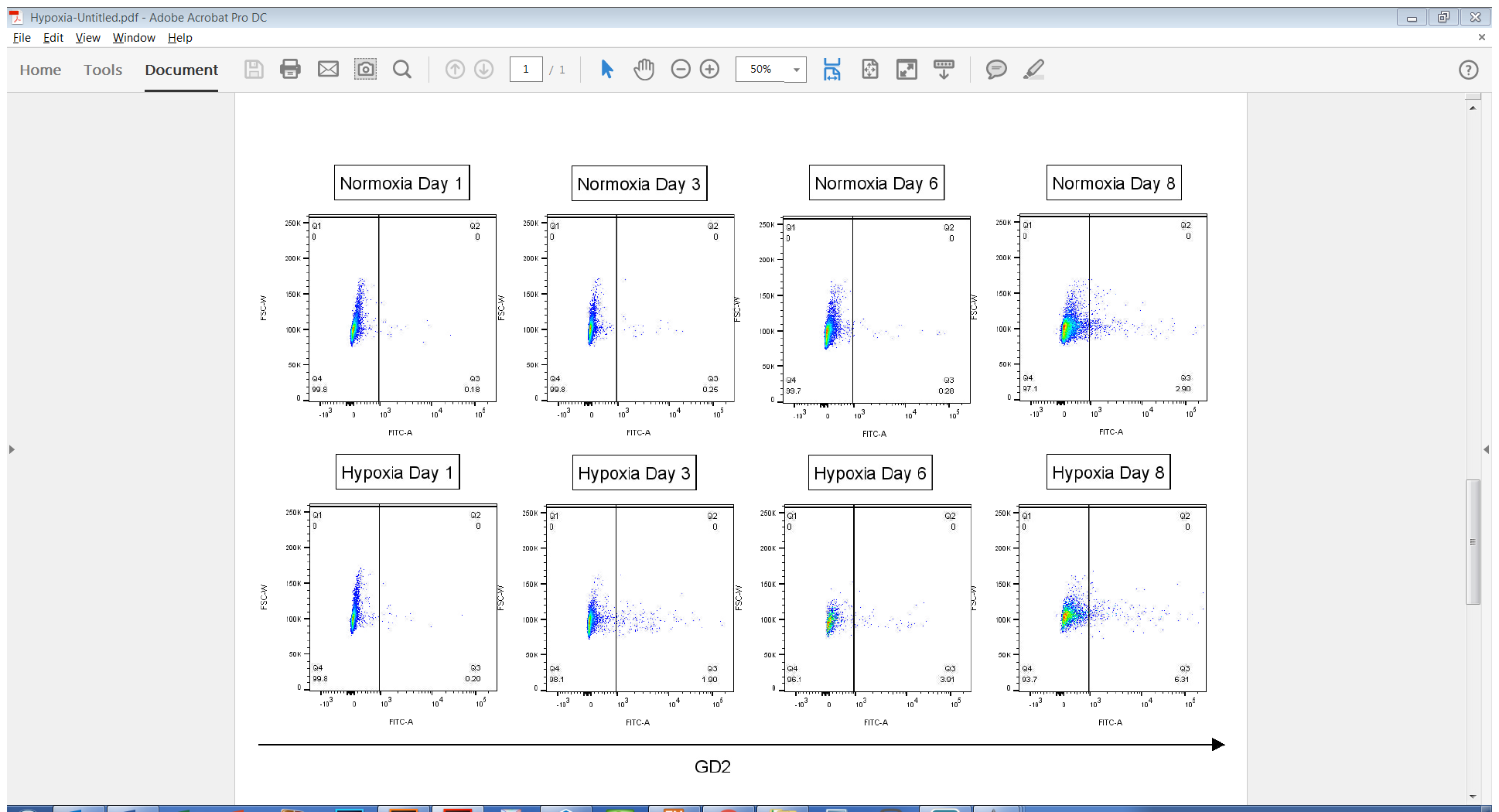


D


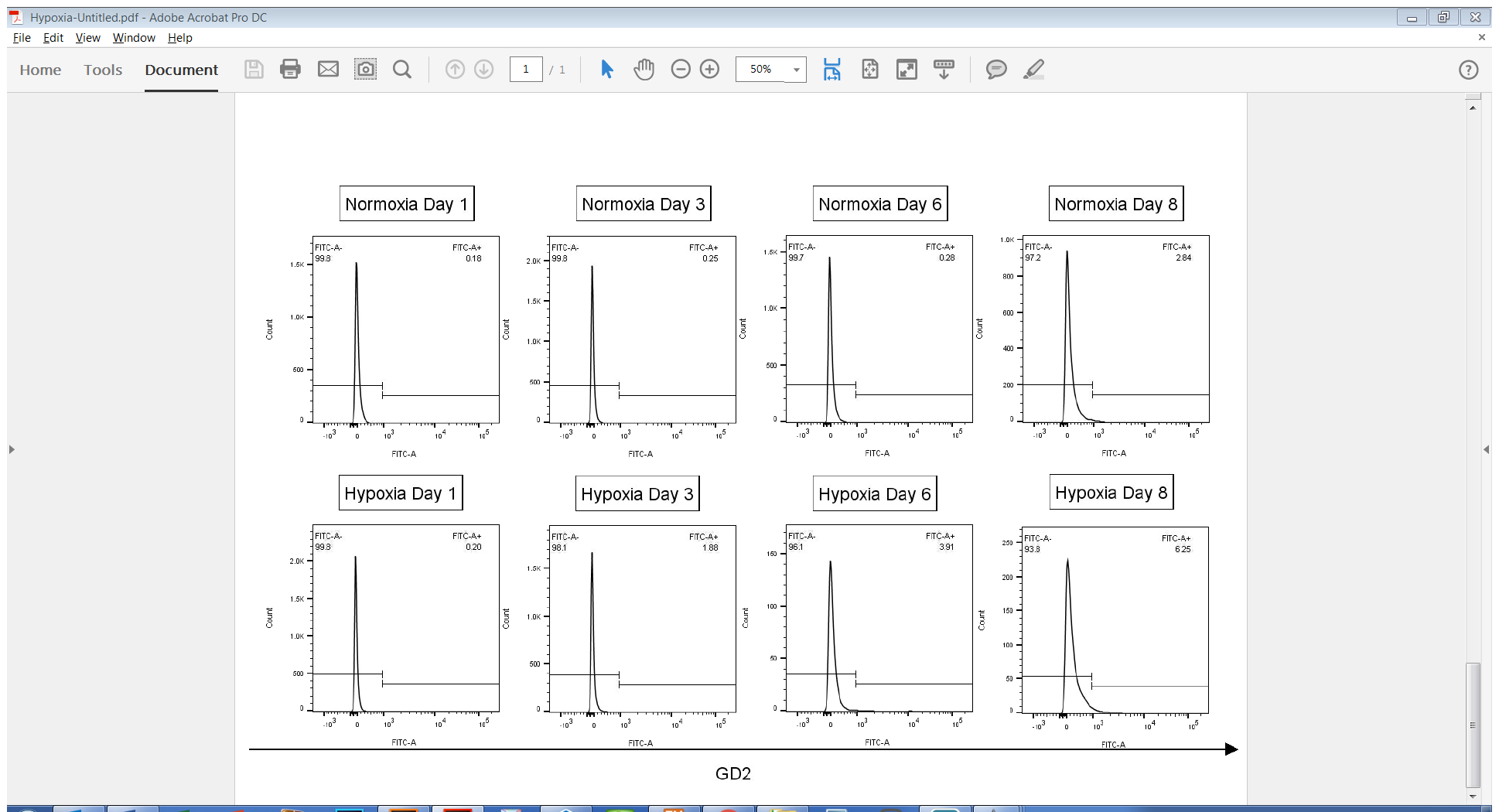


E
